# Transcriptomic profiling and regulatory pathways of cardiac resident macrophages in aging

**DOI:** 10.1007/s00018-024-05235-x

**Published:** 2024-05-20

**Authors:** Guofang Xia, Simeng Zhu, Yujia Liu, Jingwei Pan, Xiaoqing Wang, Chengxing Shen, Ailian Du, Congfeng Xu

**Affiliations:** 1 Department of Cardiology, Shanghai Jiao Tong University School of Medicine Affiliated Sixth People’s Hospital, Shanghai, China; 2 Department of Neurology, Tongren Hospital, Shanghai Jiao Tong University School of Medicine, Shanghai, China; 3 Department of Cardiology, Sichuan Academy of Medical Sciences and Sichuan Provincial People’s Hospital (SAMSPH), Chengdu, China

**Keywords:** Aging, Macrophages, Cardiac resident macrophages, Transcriptomic profiling, Smart-Seq

## Abstract

Cardiovascular diseases are an array of age-related disorders, and accumulating evidence suggests a link between cardiac resident macrophages (CRMs) and the age-related disorders. However, how does CRMs alter with aging remains elusive. In the present study, aged mice (20 months old) have been employed to check for their cardiac structural and functional alterations, and the changes in the proportion of CRM subsets as well, followed by sorting of CRMs, including C-C Motif Chemokine Receptor 2 (CCR2)^+^ and CCR2^–^ CRMs, which were subjected to Smart-Seq. Integrated analysis of the Smart-Seq data with three publicly available single-cell RNA-seq datasets revealed that inflammatory genes were drastic upregulated for both CCR2^+^ and CCR2^–^ CRMs with aging, but genes germane to wound healing were downregulated for CCR2^–^ CRMs, suggesting the differential functions of these two subsets. More importantly, inflammatory genes involved in damage sensing, complement cascades, and phagocytosis were largely upregulated in CCR2^–^ CRMs, implying the imbalance of inflammatory response upon aging. Our work provides a comprehensive framework and transcriptional resource for assessing the impact of aging on CRMs with a potential for further understanding cardiac aging.

**Supplementary Information:**

The online version contains supplementary material available at 10.1007/s00018-024-05235-x.

## Introduction

As life expectancy increases and the aged population (> 65 years old) grows, age-related disorders, especially cardiovascular diseases, pose a grave global challenge to public health and a substantial economic burden on healthcare system [1]. Age-related cardiac alterations, such as cardiac hypertrophy, myocardial fibrosis, diastolic dysfunction and cardiac reserve capacity decline, progressively increase the vulnerability of individuals to cardiovascular diseases [2]. It is commonly accepted that immuno-senescence and consequent inflammaging play a critical role in aging and age-associated disorders, including cardiac pathology. However, how senescent immune cells contribute to cardiac function and pathology with aging still remains unclear.

As prevalent immune cells in heart, cardiac resident macrophages (CRMs) have been demonstrated to be pivotal for cardiac development, homeostasis, and function such as electrical conduction. They also facilitate wound healing and cardiac repair after injury such as myocardial infarction. Distinct subsets of cardiac macrophages have been identified in the heart, and they possess particular roles in various pathophysiological conditions [3, 4]. Ontogenically, CRMs consist of C-C Motif Chemokine Receptor 2 (CCR2)^–^ subset, which is yolk-sac-derived, and CCR2^+^ subset, which is monocyte-derived [5]. CCR2^–^ CRMs are critical for cardiac homeostasis, and undergo gradual loss upon injury and aging, while CCR2^+^ CRMs are recruited and contribute to resident macrophages [6]. Recently, single-cell RNA sequencing (sc-RNAseq) revealed that both CCR2^–^ and CCR2^+^ subsets of CRMs are enriched in pathways of wound healing, angiogenesis, and vasculature development in cardiac development and cardiovascular diseases [7]. In addition, CCR2^–^ subset is mainly involved in pathways of cellular transport and endocytosis, while CCR2^+^ subset implies more in pathways concerning immune effector processes [8].

Although CRMs have been studied physiologically and pathologically, their roles in aging have not been fully explored [9–11]. Actually, both pros and cons of macrophage-mediated inflammation with aging have been referred to in literature [12]. In this study, we sorted cardiac macrophages, both CCR2^–^ and CCR2^+^ subsets, from aged mice (20 months old), and subjected them to Smart-Seq, and we then integrated our phenotypic, flow cytometric and multicellular Smart-sequencing data to create a comprehensive transcriptional framework of CRMs in aged hearts. Our work provides transcriptomic profiling of CRM populations in aged mice, contributing to establishing macrophage transcriptomic resource, and to provoke further research on the application of macrophage regulation for aging-related diseases.

## Materials and methods

### Animals

Female C57BL/6J young (3 months old, *n* = 10) and aged (20 ± 0.5 months old, *n* = 14) mice were used in this study. All mice were obtained from and kept in the specific pathogen-free facility of Shanghai Model Organism Center (Shanghai, China). All mice were fed with normal chow diets and sterile water ad libitum.

### FACS sorting of CRMs

Mice were anesthetized with 2.5% isoflurane and intracardially perfused with cold PBS to exclude blood. Hearts were harvested, and left ventricles were separated for further procedures. The left ventricle tissue was minced into ~ 1mm^3^ pieces with fine scissors and digested in PBS with 450 U/mL type I collagenase (Worthington Biochemical Corporation, Lakewood, NJ, USA), 450 U/mL type II collagenase (Worthington Biochemical Corporation), 150 U/mL type IV collagenase (Worthington Biochemical Corporation) and 60 U/mL DNase I (Worthington Biochemical Corporation) for 60 min at 37℃ with gentle agitation. Subsequently, the hearts were repeatedly aspirated with a pipette, passed through a 70-µm cell strainer, and washed with PBS. The single cell suspensions were incubated with anti-CD16/32 antibody (clone 93, eBioscience) on ice for 15 min. Subsequently, the cells were washed once and incubated with the following antibodies on ice for 30 min: anti-CD11b-BV605 (clone M1/70, BD Bioscience), anti-CX3CR1-PE-cy7 (clone SA011F11, Biolegend) and anti-CCR2-BV421 (clone SA203G11) antibodies. All labeled cells were passed through a FACSAria™ III Cell Sorter (BD Biosciences, US) and, after proper gating, at least 5000 CRMs were sorted for further sequencing. Data were analyzed using FlowJo software (TreeStar Inc. US).

### Sample preparation and Smart-Sequencing

Total RNA was extracted using TRI Reagent (Sigma Aldrich, US) from FACS-sorted CRMs. Sequencing libraries were prepared using the Smart-Seq v4 Ultra Low Input RNA Kit from Clontech (Japan). The library preparation procedures involved the following steps: (1) reparation of cDNA library; (2) Purification and size selection of cDNA fragments (150–300 bp); (3) Preparation of sequencing-ready library (by incorporating i5 and i7 adaptors using the PCR method); (4) Quality control of the library (by assessing its size and concentration using Agilent 2100 Bioanalyzer); (5) Sequencing of the library performed on Illumina platforms using a 2 × 150 bp paired-end sequencing protocol.

### Bioinformatic analysis for cardiac macrophages Smart-Seq

The bioinformatic analysis pipeline of Smart-Seq data was designed according to the guideline written by Conesa A, et al. [13]. The read quality for raw reads was performed by FastQC and poor-quality sequences and adaptors were trimmed with Cutadapt software. Reads were mapped to reference mouse genome and transcriptome (Ver. 101) with HISAT2. The assembly of mapped reads was conducted using StringTie. Differential expression analysis was performed by *DESeq2* (v 3.18) with log2 (fold change) set to > 2 or < -2, FDR < 0.05. Principal coordinate analysis of the transformed data of all samples was conducted with plotPCA function in *DESeq2*. The relevance between different samples was compared with pearson correlation coefficient. Heatmaps of DEGs were Hierarchically clustered using hclust function in stats (v 3.6.2). For performing principal component analysis (PCA), raw counts were normalized, transformed using vld method and visualized using PCAplot function in *DESeq2* package. Gene ontological (GO) analysis was performed and visualized with clusterProfiler (version 3.0.4). Pathway and transcription factors enrichment was performed with g: Profiler [14] and the OmicStudio tools at https://www.omicstudio.cn/tool. The alternative splicing analysis was performed using OmicStudio tools and enriched for GO process using g: Profiler.

### Integrated bioinformatic analysis of single-cell sequencing

The scRNA-seq datasets included in this analysis were obtained from ArrayExpress database (E-MTAB-7869 and E-MTAB-13,093) [15, 16] and Tabula Muris Senis [17]. The count data was cleared according to the following criteria: unique molecular identifiers between 200 and 500; and < 15% of mitochondrial genes to filter low-expression cells and for quality control. After the quality-control procedure, all the features were log-normalized and scaled by multiplying 10,000 as scale factor using Seurat v5 [18]. To integrate the three scRNA-seq datasets, we used a strategy that combined canonical correlation analysis and mutual nearest neighbors to identify the cross-sample gene pairs as “anchors”, which corrects for batch effect between datasets [19]. The top 2000 genes with the highest variance were used to apply PCA, and the first 15 dimensions revealed from PCA were used for Uniform Manifold Approximation and Projection (UMAP) and Leiden clustering. *Wilcoxon* rank-sum test (with min.pct = 0.25, logfc. threshold = 0.25) was performed to identify differentially expressed genes (DEGs) among clusters (also known as cluster markers). Single-cell trajectory analysis for macrophages was performed with *Monocle 3* (v.1.3.5) algorithm (https://cole-trapnell-lab.github.io/monocle3), and the trajectory was built on the UMAP from Seurat tool. To identify the DEGs between young and aged murine CRMs, pseudo-bulk RNA analysis was conducted by aggregating the counts for gene expression in all CRMs from each sample to form the pseudo-bulk count matrix and DEGs were generalized with *DESeq2* method [20].

### Quantitative polymerase chain reaction (qPCR)

Mice were anesthetized using 2.5% isoflurane and intracardially perfused with pre-cold PBS to exclude intravascular blood. Upon sacrifice, heart samples were harvested and frozen with liquid nitrogen for extracting total RNA using TRI Reagent (Sigma Aldrich). Reverse transcription was processed with HiScript II Reverse Transcriptase (Vazyme, Jiangsu, CN) according to the manufacturer’s instructions. Real-time PCR analysis was conducted on a Light Cycler 96 Real-Time system (Roche) for 45 cycles, using a SYBR qPCR Master Mix (Vazyme). qRT-PCR for *Cfb*, *C4b*, *C6*, *Cxcl13*, *Ccl8*, *Grb2*, *Rac1*, *Rhoh*, *Nr2c2*, *Ccl24*, *Igf1* was performed using the primer pairs in Suppl. Table 1.

### Histology

Mice were anesthetized with 2.5% isoflurane and sacrificed by exsanguination. After intracardially perfusing with cold PBS, heart samples were collected, fixed with 4% PFA, embedded in paraffin, and cut into 4 μm sections. Hematoxylin-eosin staining was performed on paraffin-embedded sections at the papillary level to determine the heart cross-sectional area. Masson’s Trichrome staining was performed using Masson dye solution set (Servicebio, Hubei, CN) to determine the fibrosis area of the hearts. Wheat Germ Agglutinin (WGA)-FITC (Sigma, US) was used to stain the cardiomyocyte membranes to quantify the size of myofibers. For immunohistochemical (IHC) assay, paraffin-embedded heart sections were incubated with anti-mouse F4/80 antibody (Servicebio, Hubei, CN) overnight at 4℃ and detected with anti-rabbit HRP-conjugated secondary antibody. All histology images were acquired by Olympus BX53 microscopy (Olympus Corporation, Tokyo, Japan) and analyzed with ImageJ (Version 1.54 h, NIH).

### Echocardiography

Transthoracic echocardiography was performed using a Vevo 3100 instrument (VisualSonics, Toronto, Canada) with a 55-MHz ultrasound transducer. The mice were shaved on the chest, anesthetized with 2% isoflurane, and fixed on the echo pad in a supine position. Two-dimensional images were recorded in the left ventricular long-axis planes at an appropriate position and the heart rate at 500–550 bpm. M-mode was used to measure the left ventricular anterior wall; diastole and left ventricular posterior wall; diastole.

LV Mass was calculated as

1 $$LV Mass=1.053\times \left[\right(LVID;d+LVPW;d+IVS;d{)}^{3}-LVID;{d}^{3}]$$.

Ejection fraction was calculated as

2 $$EF=100\times \left(\frac{LV{\hspace{0.17em}}Vo{l}_{d}-LV{\hspace{0.17em}}Vo{l}_{s}}{LV{\hspace{0.17em}}Vo{l}_{d}}\right)$$

Stroke volume was calculated as

3 $$SV=LV{\hspace{0.17em}}Vo{l}_{d}-LV{\hspace{0.17em}}Vo{l}_{s}$$

The apical four-chamber view was captured and the peak flow velocities during early diastole (E wave) and late diastole (A wave) across the mitral valve were calculated. The measurements were averaged over three consecutive cardiac cycles.

### Statistical analysis

All data are presented as mean ± SD. Statistical comparisons were performed using GraphPad Prism v9.5.0 software. The unpaired Student’s *t* test was used for all statistical analyses in this study. *p* value less than 0.05 is considered statistically significant.

## Results

### Age-related accumulation of macrophages in murine hearts

**Fig. 1:**
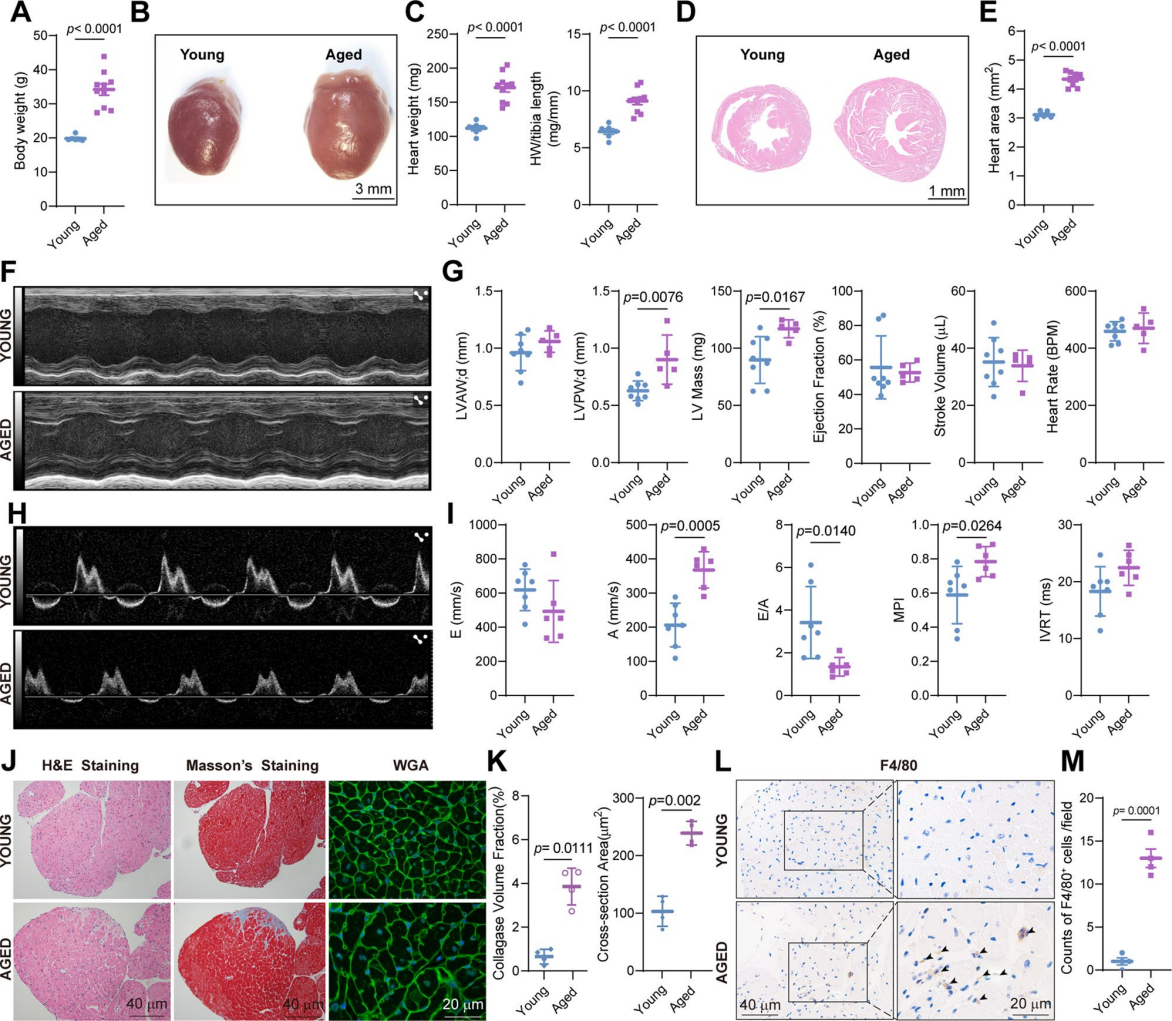
Phenotypic alterations in aged mouse heart **A**, Quantification of body weight. Data are shown as mean ± SD (*n* > 6 mice per group). **B**, Representative image for the overall heart size of young (12-week old) mice and aged (20-month old) mice. **C**, Quantification of heart weight and heart weight-to-tibia length ratio. Data are shown as mean ± SD (*n* > 6 mice per group). **D**, Representative H&E staining of the cross section at the papillary muscles level in young and aged mice hearts. **E**, Quantification of heart area. Data are shown as mean ± SD (*n* > 5 mice per group). **F**, Representative echocardiographic images of the left ventricular long-axis planes M-mode view for young and aged mice. **G**, Quantification of LVAW;d, LVPW;d, LV Mass, Ejection fraction, Stroke volume and Heart Rate. Data are shown as mean ± SD (*n* > 5 mice per group). LVAW;d, Left ventricular anterior wall; diastole. LVPW;d, Left ventricular posterior wall; diastole. **H**, Representative image of pulsed Doppler of mitral inflow obtained from young and aged mice. **I**, Quantification of E wave, A wave, E-to-A ratio, MPI and IVRT. Data are shown as mean ± SD (*n* > 5 mice per group). MPI, myocardial performance index. IVRT, isovolumic relaxation time. **J**, Representative H&E staining, Masson’s Trichrome staining and WGA staining of young and aged mice hearts. **K**, Quantification of collagen volume fraction, cross-section area of cardiomyocytes. **L**, Representative F4/80 IHC staining of young and aged mice hearts. **M**, Quantification of macrophages is measured as the count of F4/80 positive cells per high magnification (× 400) field of view. Data are shown as mean ± SD (*n* = 4 mice per group)

To investigate the impacts of aging on the murine heart, we obtained and measured the hearts from young mice (12 weeks old) and aged mice (20 months old) (Fig. 1A). Both heart weight and heart weight-to-tibia length ratio were significantly augmented in aged mice, implying cardiac hypertrophy with aging (Fig. 1B and C), which was supported by Hematoxylin and Eosin (H&E) staining of the entire heart section (Fig. 1D and E). In addition, we evaluated the age-associated alteration in left ventricle structure and function using echocardiography, and found a significant increase in the thickness of left ventricular anterior wall and left ventricular mass in aged mice (Fig. 1F and G). Although there was no significant difference for left ventricular ejection fraction and stroke volume (Fig. 1F and G), we did observe that aged mice had decreased values of E/A ratio (Fig. 1H and I), indicating the diastolic dysfunction in aged mice. We also checked the cardiomyocyte size and collagen deposition in the heart, and found that aged mice displayed an increased cardiomyocyte cross-sectional area with WGA staining, and more evident interstitial and perivascular fibrosis in the aged mice (Fig. 1J and K). Furthermore, we detected CRM in aged murine hearts using IHC analysis for F4/80 and found a significant increase in the number of CRMs in aged murine hearts (Fig. 1L and M). Thus, we systematically characterize the phenotypic changes of murine hearts upon aging.

### CRMs undergo evident transcriptomic alteration in aging

**Fig. 2:**
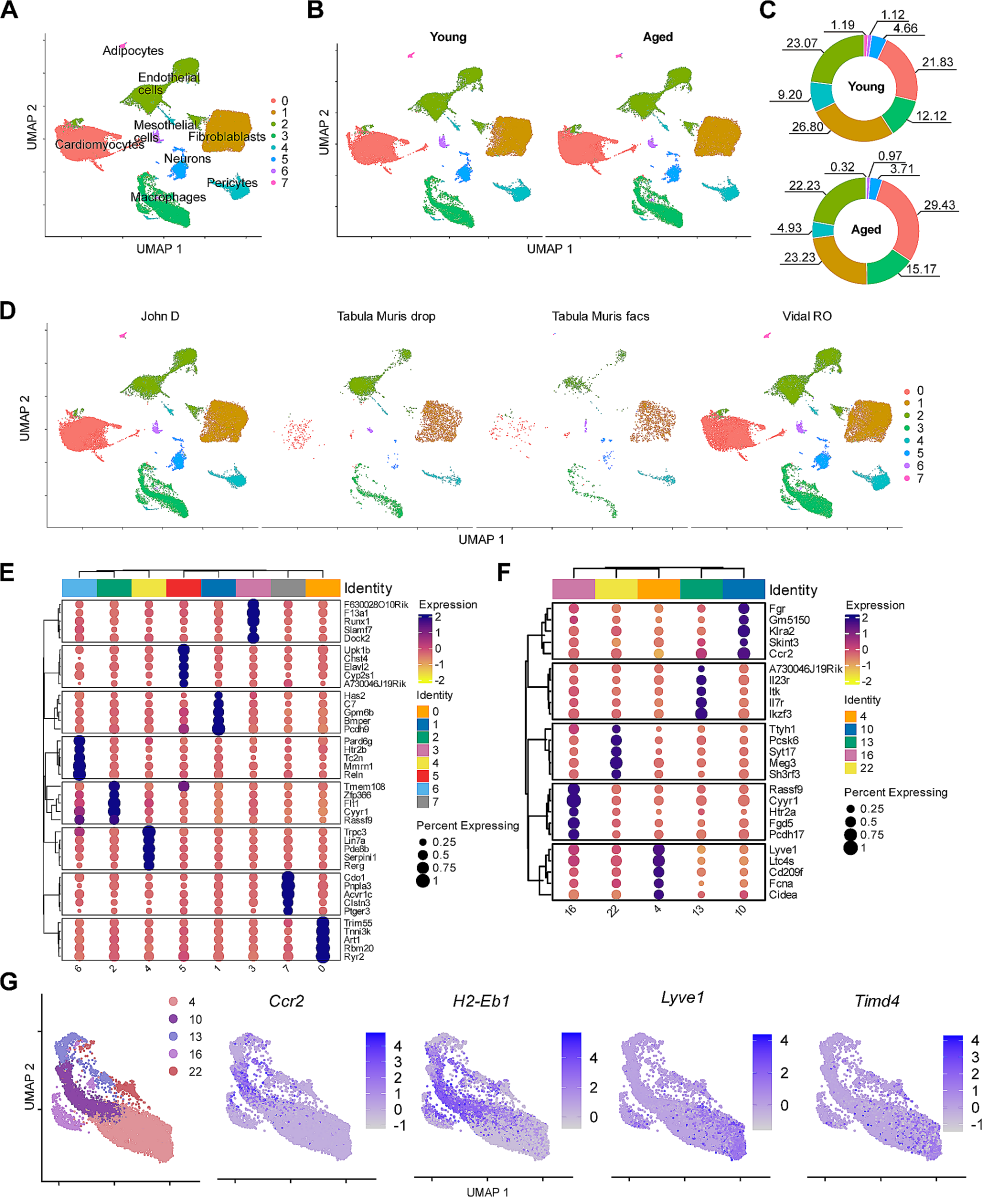
Integration of single-cell RNA-seq datasets from aged mice hearts. **A**, UMAP of cardiac cells with seurat cluster in resolution 0.05. **B**, UMAP split by young and aged condition. **C**, Quantification of the relative abundance of each cluster under young and aged conditions. **D**, UMAP showing the distribution of the three datasets. **E**, Heatmap of the top 10 differentially expressed genes in each cluster (logFC threshold = 0.25, min.pct = 0.25, adjusted p-value < 0.05). **F**, Heatmap of the top 5 differentially expressed genes in the CRMs sub-clusters with resolution 0.5 (logFC threshold = 0.25, min.pct = 0.25, adjusted p-value < 0.05). **G**, UMAP of CRMs sub-clusters with a resolution of 0.5 and Featureplots of CRMs’ marker genes

**Fig. 3:**
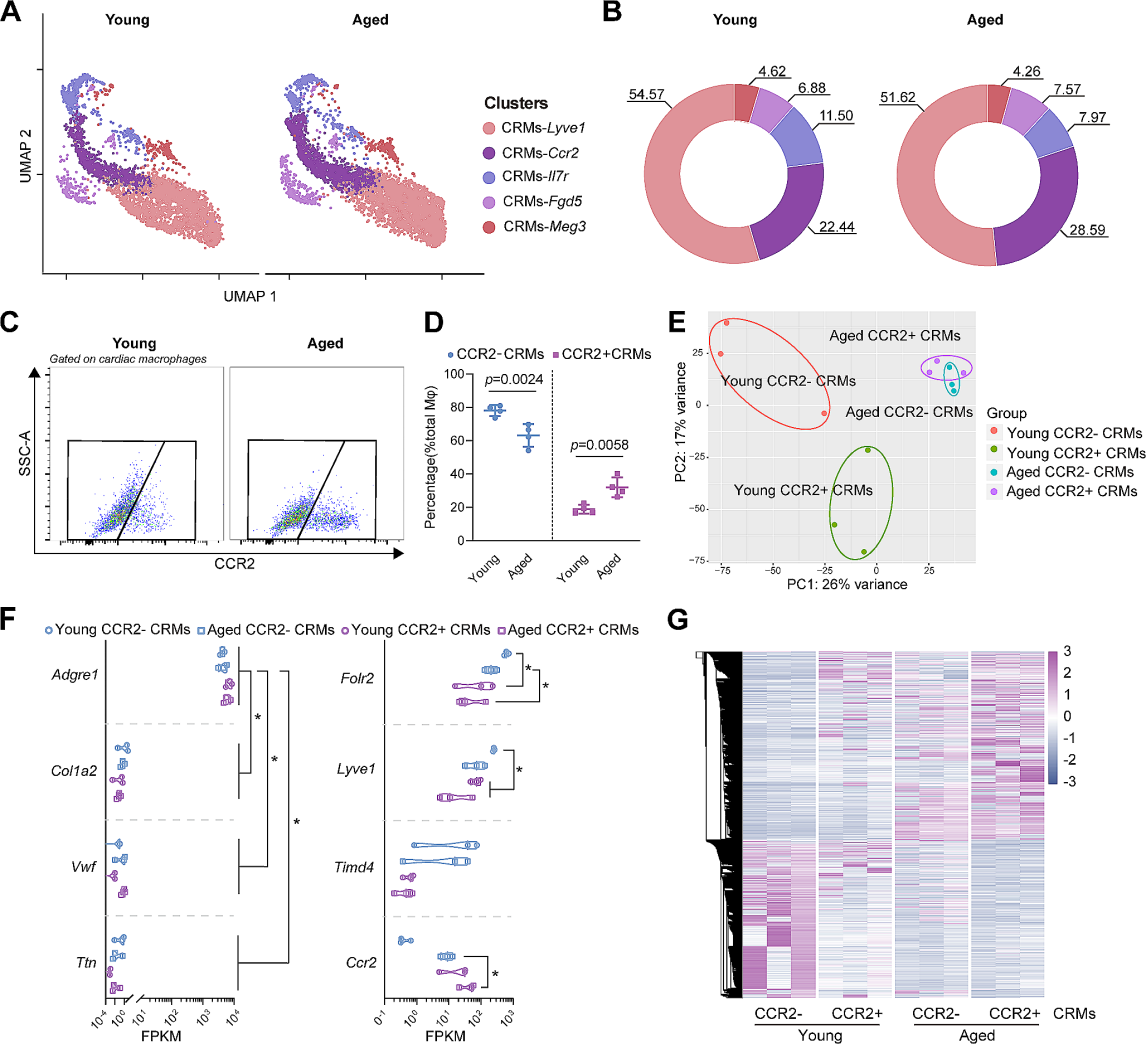
The sub-clusters and quantity of CRMs in aging. **A**, UMAP of the macrophages in young and aged mice heart. UMAP, Uniform Manifold Approximation and Projection. **B**, Quantification of the relative abundance of each cluster under young and aged conditions. **C**, Representative flow cytometric plots of CRMs in young and aged mice. **D**, Quantification of the proportion of CCR2^–^ and CCR2^+^ CRMs. Data are shown as mean ± SD (*n* = 4 mice per group). **E**, PCA plot of CCR2^–^ and CCR2^+^ CRMs across young and aged mice. **F**, Purity of isolated CRMs based on expression of known cardiac cell type–specific markers. Data are shown as mean ± SD (*n* = 3 mice per group). *p < 0.05. FPKM, fragments per kilobase of transcript per million fragments mapped. **G**, Heatmap of significantly differentially expressed genes with log2 fold change > 2 and *p* value < 0.05

To determine the cellular heterogeneity of the CRMs in aged heart, we integrated three publicly available scRNA-seq datasets conducted on young and aged murine hearts [15–17], which included a total of 66,985 cells derived from the hearts of 9 young and 13 aged mice. With Seurat v5 to mitigate batch effects [19], the cardiac cells were clustered into 8 subpopulations using unsupervised methods and were annotated as major cell types in the heart (Fig. 2A–E). Then we went further to recluster the macrophages into 5 clusters (Fig. 2F and G). Although there was no additional subpopulation of CRMs in aged mice, we observed an increase in counts of CRMs-*Ccr2* subclusters and a decrease of CRMs-*Lyve1* CRMs in aged mice (Fig. 3A and B). Taking advantage of flow cytometry, we noticed that CCR2^+^ cardiac macrophages significantly expanded with age in the heart, while CCR2^–^ cardiac macrophages shrank (Fig. 3C and D).

To elucidate the transcriptomic profiles of CRMs upon aging, we employed fluorescence-activated cell sorter (FACS) to sort both CCR2^–^ and CCR2^+^ cardiac macrophages, two distinct subpopulations, from hearts of mice (Fig. 3C, as well as Suppl. Figure 1A). Most sorted cells were viable before total RNA extraction, and more than 5000 cells per sample were subjected to Smart-sequencing. RNA-seq libraries were prepared from each group, and 25,131 unique transcripts were available for analysis. Analysis of cell type–specific transcripts confirmed that our purified cell populations were highly enriched for macrophage-specific gene markers. CCR2^–^ CRMs highly expressed TLF^+^CRMs gene markers, such as *Timd4, Lyve1 and Folr2*, while CCR2^+^ CRMs exhibited high expression of the *Ccr2* gene (Fig. 3F). Through principal component analysis and hierarchical clustering heatmap, CRMs from young and aged mice were segregated into two distinct patterns, indicating that CRMs from young and aged mice possessed unique gene expression profiles (Fig. 3E, G, Suppl. Figure 1B and C).

### CCR2^−^ and CCR2^+^ macrophages possess distinct transcriptomic profiles in aged heart

**Fig. 4:**
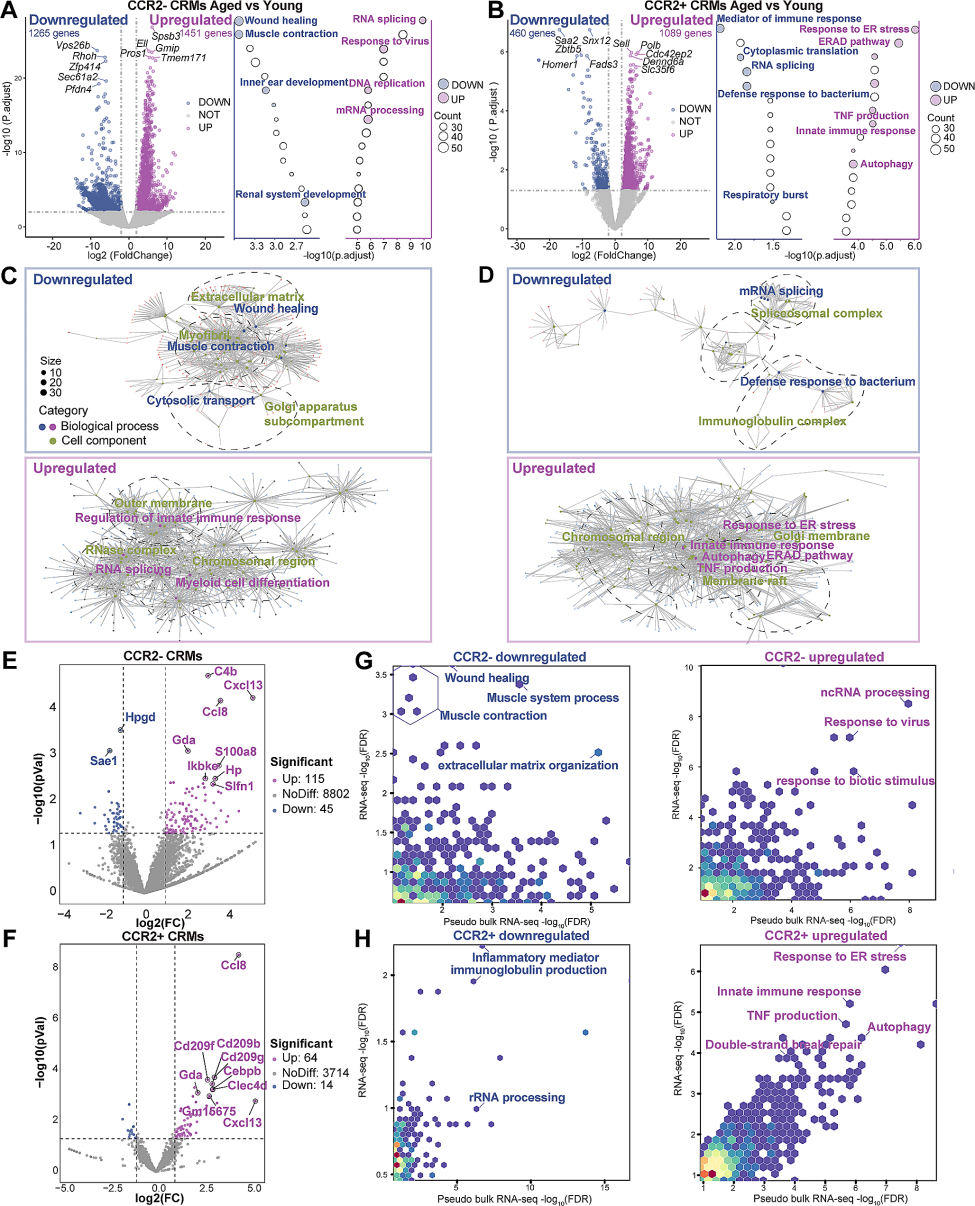
Integrated analysis of Smart-Seq and pseudo-bulk RNA analysis for sc-RNA seq. **(A** and **B)**, Left: Volcano plots portray DEGs of aged versus young mice in CCR2^–^ CRMs **(A)** or CCR2^+^ CRMs **(B)**. DEGs with *p* value < 0.05 are marked in shades of blue (downregulated) or shades of purple (upregulated). Right: Enriched gene ontologies for the DEGs of aged versus young mice in CCR2^–^ CRMs **(A)** or CCR2^+^ CRMs **(B)**. Ontologies are ranked based on –log (*P* adjust) from top to bottom. Representative gene ontologies are highlighted with colored circles and names are displayed. **(C** and **D)**, The cnetplot depicts the linkages of the five most enriched biological processes and cellular components in CCR2^–^ CRMs **(C)** and CCR2^+^ CRMs **(D)**. Biological processes are marked with shades of purple or blue and cellular components are marked with shades of green. (**E** and **F)**, Volcano plots portray DEGs of aged versus young mice in CCR2^–^ CRM **(E)** or CCR2^+^ CRMs **(F)** from pseudo-bulk RNA analysis for integrated scRNA-seq dataset. DEGs with *p* value < 0.05 are marked in shades of blue (downregulated) or shades of purple (upregulated). (**G** and **H)**, Scatterplot showing biological processes enriched for CCR^–^ CRMs **(G)** or CCR2^+^ CRMs **(H)** in Smart-Seq and pseudo-bulk RNA analysis. −log10(FDR) is plotted for pseudo-bulk RNA analysis (x axis) and Smart-Seq (y axis). Yellow indicates high and blue indicates low gene set density

Using *DESeq2* method, 4265 differentially expressed genes among groups were determined based on criteria of fold change > 4 and *p* value < 0.05. CCR2^–^ CRMs had 1451 genes upregulated and 1265 genes downregulated from the aged mice, while CCR2^+^ CRMs had 1089 genes, and 460 genes, respectively (Fig. 4A and B), indicating that both CCR2^–^ and CCR2^+^ CRMs are highly transcriptionally active. Subsequently, we evaluated the biological processes of macrophage subsets and conducted Gene Ontology (GO) analysis and transcriptional network analysis for up- and down-regulated genes in aged heart (Fig. 4A–D). In CCR2^–^ CRMs, down-regulated genes were mainly related to wound healing, and muscle contraction in aging, while in CCR2^+^ CRMs, down-regulated genes were related to mediator of immune response and respiratory burst. In CCR2^–^ CRMs, up-regulated genes were related to innate immune response, and response to virus, while in CCR2^+^ CRMs, up-regulated genes were related to innate immunity, ER stress and autophagy (Fig. 4C and D). Visualization using the cnetplot function in the enrichplot package showed that inflated processes tend to localize in outer membrane in CCR2^–^ CRMs, while they were in the membrane raft and Golgi membrane in CCR2^+^ CRMs (Fig. 4C and D). To further investigate the transcriptomic regulation network of CRMs, we enriched the transcription factors for major biological processes and identified eleven main transcription factors for these biological processes (Suppl. Figure 2). Analysis based on above mentioned scRNA-seq dataset [20] also showed similar biological processes to our data, e.g., downregulated wound healing, muscle contraction in CCR2^–^ CRMs, downregulated mediator of immune response in CCR2^+^ CRMs; upregulated response to virus in CCR2^–^ CRMs, upregulated innate immunity, ER stress, and autophagy in CCR2^+^ CRMs (Fig. 4G and H).

**Fig. 5:**
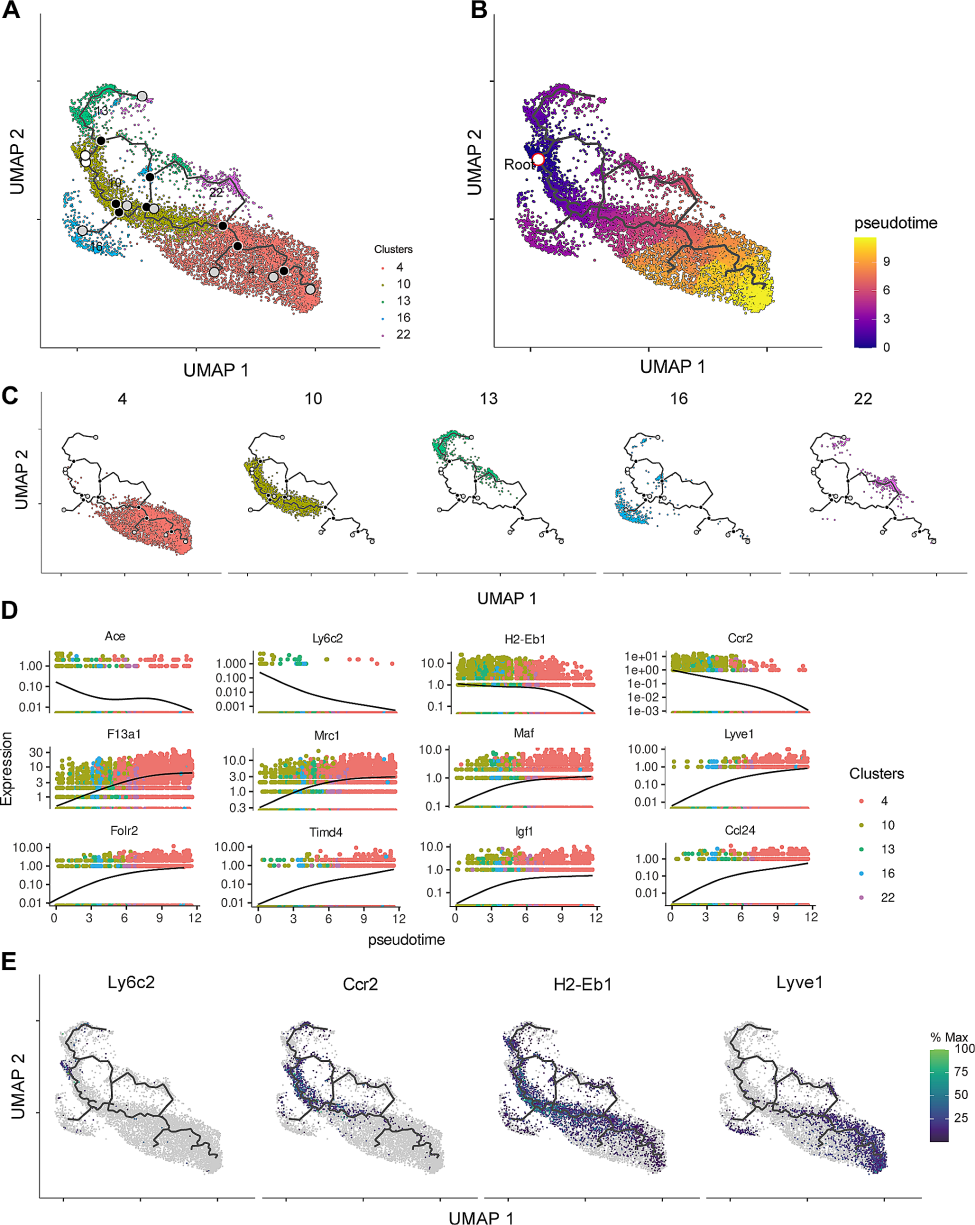
Pseudotime and single-cell trajectory analysis of CRMs by Monocle. **A**, Monocle analysis for the CRMs showing the activation states and differentiation trajectories. **B**, Pseudotime trajectory analysis showing the order of activation states. **C**, Pseudotime trajectory analysis showing the distribution of each Seurat clusters. **D**, Dynamic changes in gene expression on the pseudotime axis. **E**, Cluster-defining gene expression on pseudotime map

To explore possible cause of the age-related transcriptomic alteration in aged CRMs, we reconstructed the single-cell trajectory graph for CRMs using *Monocle 3* algorithm (Fig. 5). This analysis revealed a continuous differentiation path between CRMs-*Ccr2* and CRMs-*Lyve1*. Appointing the *Ly6c2*^high^ cells as a start point, a possible differentiation trajectory from CRMs-*Ccr2* to CRMs- *Lyve1* was sketched in aged heart (Fig. 5A-C). Plotting the macrophage marker genes expression as a function of pseudotime, we found that *Ly6c2*, *H2-Eb1* and *Ccr2* were decreased, while *Lyve1*, *Folr2*, *Timd4*, *Igf1* and *Ccl24* were increased with pseudotime(Fig. 5D and E).

In addition to differential expression regulation, alternative splicing of precursor mRNAs is another crucial regulatory mechanism for eukaryotic protein diversity [21]. Given the notably enriched RNA splicing process in CRMs during aging, we analyzed the alternative splicing events that occurred in the CRMs from aged heart, and identified 2247 significantly altered splicing events in CCR2^–^ CRMs and 2025 in CCR2^+^ CRMs during aging (Suppl. Figure 3 A and D). GO enrichment analysis of the differential alternative splicing events in CRMs revealed that both CCR2^–^ and CCR2^+^ CRMs are enriched for biological processes including protein catabolic process and immune system process (Suppl. Figure 3B and E). These biological processes are represented by the top-ranked exon skipping occurrence in *Csf2a, Ler3ip1, Ndufa7* and *Nrros* (Suppl. Figure 3 C and F).

In summary, our results reveal that both CCR^–^ and CCR2^+^ CRMs develop pro-inflammatory and stress response transcriptional profiling, and upregulate the RNA splicing-related gene transcription during aging, while functions related to cardiac homeostasis were uniquely downregulated in CCR2^–^ CRMs.

### Inflated inflammatory cascades were activated in aging

**Fig. 6:**
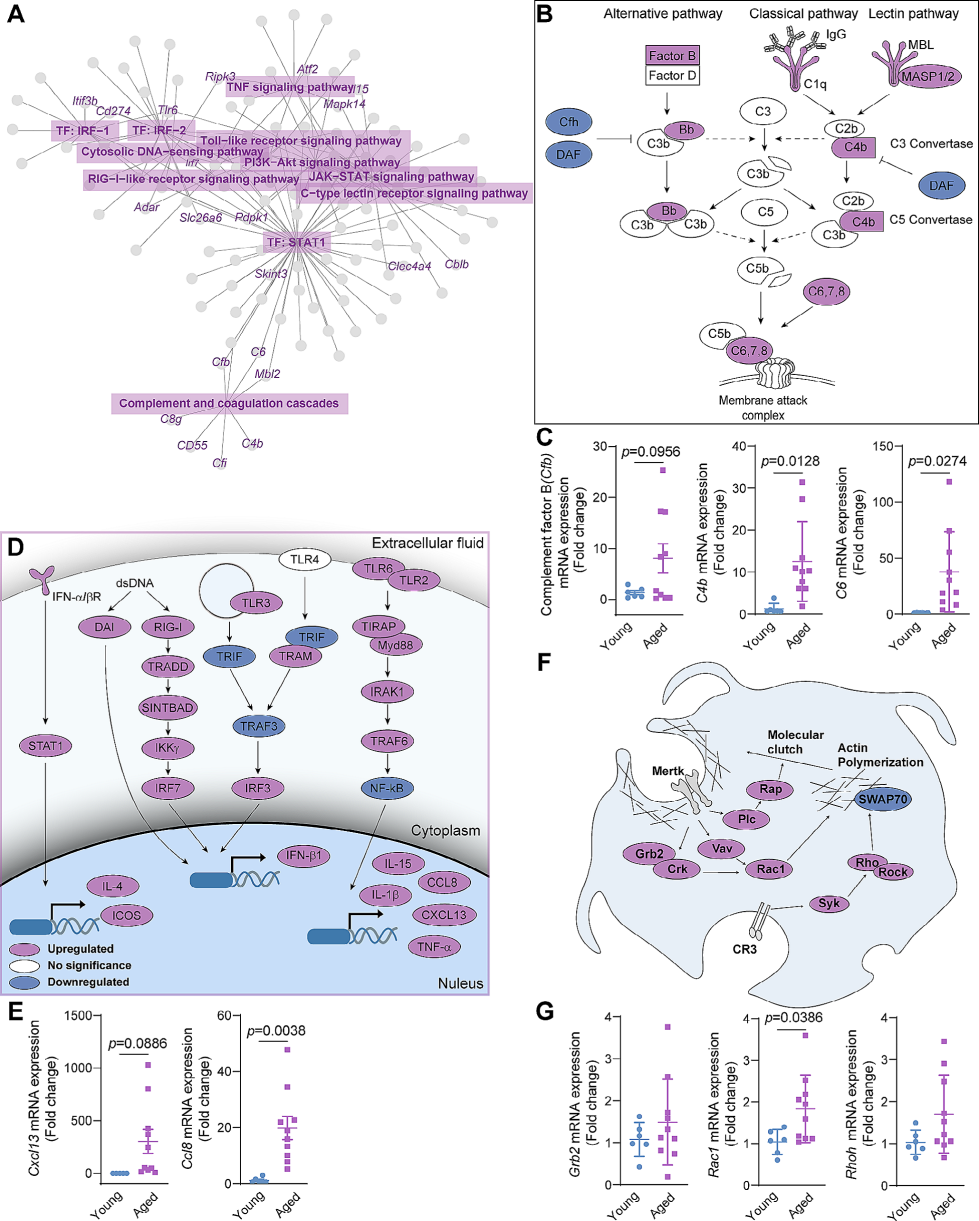
The changes of inflammation and phagocytosis process in CRMs during aging **A**, Network plot of KEGG pathway and transcription factors enriched in immune-related DEGs of aged versus young mice in CCR2^–^ CRMs. **(B, D** and **F)**, Schematic diagram of Fcg-mediated phagocytosis and CR3-mediated phagocytosis pathway **(B)**, immunity-related pathways **(D)** and complement cascade **(F)** annotated with gene expression. Genes are represented as circles and marked in shades of blue (downregulated) or shades of purple (upregulated). **(C, E** and **G)** qPCR quantification of *Grb2*, *Rac1*, *Rhoh*, *Cxcl13*, *Ccl8*, *Cfb*, *C4b* and *C6* transcription level in heart tissue from young and aged mice. Data are shown as mean ± SD (*n* ≥ 6 mice per group). *p<0.05

Given the numerous genes related to innate immune process involved in aging in CCR2^–^ CRMs, we re-analyzed the immune-related genes by KEGG pathway and transcription factor enrichment, and found that differentially upregulated genes were mainly enriched in the complement cascade, TNF signaling and cytosolic DNA-sensing pathway, all of which were several well-known pathways contributed to inflammation (Fig. 6A). We noticed that many complement components, such as factor b, Bb, were upregulated, while negative regulators, such as Cfh and DAF, were downregulated (Fig. 6B). Thus, they corporately work to push forward inflammatory response. Even more prominent changes were observed in PRR-mediated pathways, especially IRF-3/7-mediated pathways (Fig. 6D), potentially leading to chronic inflammation and virus response. We validated the upregulated genes related to complement cascade and pro-inflammatory response using qPCR, and found significant increase in *Cfb*, *C4b*, *C6*, *Cxcl13* and *Ccl8* gene expression (Fig. 6C and E). Interestingly, we found that the genes related to Fcg receptor-mediated phagocytosis and complement receptor 3-mediated phagocytosis were upregulated, while the effectors of phagocytosis were downregulated (Fig. 6F), indicating the impaired phagocytosis process in aging. The key genes in this pathway, such as *Grb2*, *Rac1* and *Rhoh*, have been validated by qPCR (Fig. 6G). Reasonably, the inflated CRM-mediated inflammation responses with aging contribute to aging and local inflammaging.

## Discussion

Mainly with diastolic dysfunction and fibrosis in aged mice, more CRM accumulation was also observed in aged murine hearts. During the process of aging, CCR2^+^ CRMs progressively replaced CCR2^–^ CRMs. Taking advantage of FACS sorting and Smart-Seq, we performed systematic analysis of unbiased transcriptomic signature and alternative splicing, and found that aged macrophages undergo a functional shift to pro-inflammatory state, represented by inflated pro-inflammatory bioprocesses, such as cytosolic DNA-sensing pathway, TNF signaling pathway, and complement cascade, under the control of transcription factor IRF3, IRF7 and NF-κB, respectively. We also observed a significant enrichment of DNA damage/repair and ER stress processes in aged CRMs, some potent driver for aging of CRMs. Thus, our study provides a comprehensive framework and transcriptional resource for assessing the impact of aging on CCR2^–^ and CCR2^+^ CRMs, which play critical roles in the pathogenesis of various cardiovascular diseases.

In the elderly, the aging heart is known to experience age-related left ventricular alterations [22]. Through elaborated phenotypic analysis, here we demonstrated there exists significant cardiac hypertrophy and cardiac fibrosis along with diastolic dysfunction in aged murine hearts. Given the “inflammaging theory” [23], it is understandable that chronic inflammation contributes to age-related cardiac changes. As a critical player for inflammation, macrophages undoubtedly undergo profound change with aging and exert crucial influence on local microenvironment. In our study, we revealed the CRMs up-regulate the pro-inflammatory cytokines, such as IL-1β and TNF-α, i.e. senescence-associated secretory phenotype (SASP), which act on cardiomyocytes, and lead to age-related cardiac hypertrophy and diastolic dysfunction [24]. In fibroblasts, CRM-derived TGF-β serves as the master activator promoting myofibroblast transdifferentiation, contributing to interstitial fibrosis and hypertrophic remodeling [25]. We observed a significant augmentation of TGF-β–TGFβR1 crosstalk between CRMs and fibroblasts in aged mice, which potentially contribute to age-related cardiac fibrosis and dysfunction. Combining the bulk transcriptional analysis of CRMs with single-cell RNA-seq, we revealed that CRMs may interact with cardiomyocytes and fibroblasts, driving cardiac age-related phenotypic alterations.

Even in CCR2^–^ CRMs, many genes involved in complement cascade and nuclear acid-sensing pattern recognition receptors pathways were upregulated. Using KEGG pathway and transcription factor enrichment analysis, we observed upregulation of the transcription factors IRF3/7 within CCR2^–^ CRMs. Both retinoic acid-inducible gene-I (RIG-I) and DNA-dependent activator of interferon regulatory factor (DAI) are intracellular DNA sensors that trigger downstream IRF transcription factors. In addition, the DNA replication/damage pathway is significantly enriched in aged CCR2^–^ CRMs. These findings support the idea that CCR2^–^ CRM-mediated inflammatory response during aging is induced by DNA damage, which is putatively initiated by telomere shortening and thus accelerates aging [25, 26]. Conversely, marked upregulation of ER stress was observed in CCR2^+^ CRMs, suggesting the critical role of ER stress for chronic inflammation in CCR2^+^ CRMs. More distinctively, multiple genes of complement system are significantly upregulated in CCR2^–^ CRMs during aging, and may serve as an amplification or initiation mechanism for local chronic inflammation in heart aging, which could lead to age-related myocardial hypertrophy and cardiac fibrosis.

In this study, we subjected FACS-sorted cardiac macrophages to Smart-seq, together with scRNA sequencing data, to provide a more refined whole-exon transcriptomic profiling. And our study found that, with aging, cardiac macrophages skew towards chronic inflammation and endoplasmic reticulum stress. This process is also accompanied by a significant number of alternative splicing events related to immune response during aging. Meanwhile, cardiac resident macrophages may exert more influence on age-related alterations, such as promoting angiogenesis and wound healing, and pave the road ahead to further understand cardiac aging.

### Electronic supplementary material

Below is the link to the electronic supplementary material.

Supplementary Material 1

## Data Availability

All sequencing data newly generated have been deposited at the NCBI’s public functional genomics data repository Gene Expression Omnibus (The accession number for the dataset is: GEO: GSE253651, processed data are available on Series record, raw data are available in SRA) and will be made available upon publication.

## Abbreviations

C4b: Complement C4b
CCL8: C-C motif chemokine Ligand 8
CCR2: C-C Motif Chemokine Receptor 2
Cfb: Complement factor b
Cfh: Complement factor h
CRMs: Cardiac Resident Macrophages
CSF2: Colony Stimulating Factor 2
CXCL13: C-X-C motif Chemokine Ligand 13
DAF: Decay Accelerating Factor for complement
DAI: DNA-dependent Activator of Interferon regulatory factor
DEGs: Differentially Expressed Genes
ER: Endoplasmic Reticulum
FACS: Fluorescence-Activated Cell Sorting
FOLR2: Folate Receptor Beta
GO: Gene ontology
GRB2: Growth factor Receptor Bound protein 2
H&E: Hematoxylin and Eosin staining
IHC: Immunohistochemistry
IVRT: Isovolumic Relaxation Time
KEGG: Kyoto Encyclopedia of Genes and Genomes
LVAW;d: Left Ventricular Anterior wall; diastole
LVPW;d: Left Ventricular Posterior wall; diastole
LYVE1: Lymphatic Vessel Endothelial hyaluronan receptor 1
MPI: Myocardial Performance Index
NDUFA7: NADH: ubiquinone oxidoreductase subunit A7
NRROS: Negative Regulator of Reactive Oxygen Species
PCA: Principal Component Analysis
qPCR: quantitative Polymerase Chain Reaction
RAC1: RAC family small GTPase 1
RhoH: Ras homolog family member H
RIG-I: Retinoic acid-Inducible Gene-I
scRNA-seq: single-cell RNA sequencing
SASP: Senescence-associated Secretory Phenotype
TGF-β: Transforming Growth Factor beta
TIMD4: T cell Immunoglobulin and Mucin Domain containing 4
TNF: Tumor Necrosis Factor
UMAP: Uniform Manifold Approximation and Projection
WGA: Wheat Germ Agglutinin
