# Supplementary material for: Transcriptomic profiling and regulatory pathways of cardiac resident macrophages in aging
Source: Cell Mol Life Sci. 2024 May 20;81(1):220. doi: 10.1007/s00018-024-05235-x (PMC11102896; doi:10.1007/s00018-024-05235-x)
Supplement: Supplementary file 1 — Supplementary Material 1 [file 18_2024_5235_MOESM1_ESM.pdf]

Supplementary Material

Transcriptomic profiling and regulatory pathway of resident cardiac macrophages in aging

Guofang Xia[1], Simeng Zhu[1], Yujia Liu[2], Jingwei Pan[1], Xiaoqing Wang[3], Chengxing Shen[1], Ailian Du[2] and Congfeng Xu[1]

[1] Department of Cardiology, Shanghai Jiao Tong University School of Medicine Affiliated Sixth People's Hospital, Shanghai, China

[2] Department of Neurology, Tongren Hospital, Shanghai Jiaotong University School of Medicine, Shanghai, China

[3] Department of Cardiology, Sichuan Academy of Medical Sciences and Sichuan Provincial People's Hospital (SAMSPH), Chengdu, China

***Correspondence:**

Congfeng Xu (cxu@shsmu.edu.cn)

Supplementary Material

Supplementary Table 1. Primers used for qPCR

Targeted genes		Primer sequencing (5' to 3')
C4b	F'	ACTTCAGCAGCTTAGTCAGGG
	R'	GTCCTTTGTTTCAGGGGACAG
C6	F'	TTTGCTGGTCATGCTGATTGA
	R'	TCTGGTCTCCTGCTTGATACAA
Grb2	F'	CCCTGTCCGTCAAGTTTGGAA
	R'	GGCATCTGTTCTATGTCCCGTAA
Rac1	F'	GAGACGGAGCTGTTGGTAAAA
	R'	ATAGGCCCAGATTCACTGGTT
Rhoh	F'	CTACAAACCCACGGTGTACGA
	R'	CCGGATACTTCTGAAGGCGT
Nr2c2	F'	GACTCTGCGGTAGCCTCAC
	R'	AGGATGAACTGCTGTTTAGAGGA
Cfb	F'	GGAAGGAGGTGTACATCAAGAA
	R'	TGTAGAAGAGAGAAGCTTTGCA
Cxcl13	F'	TTGTGATCTGGACCAAGATGAA
	R'	GACTTTTGCTTTGGACATGTCT
Ccl8	F'	GAATCAACAATATCCAGTGCCC
	R'	TTGAGACTTCTGGTCAAGGATC
Ccl24	F'	AAAATTCCAGAAAACCGAGTGG
	R'	GGGGTCAGTACAGATCTTATGG
Igf1	F'	GAGGGGCTTTTACTTCAACAAG
	R'	TACATCTCCAGTCTCCTCAGAT
18s	F'	ACCGCAGCTAGGAATAATGGA
	R'	CAAATGCTTTCGCTCTGGTC

F’: Forward; R’: Reverse.

Supplementary Figure 1

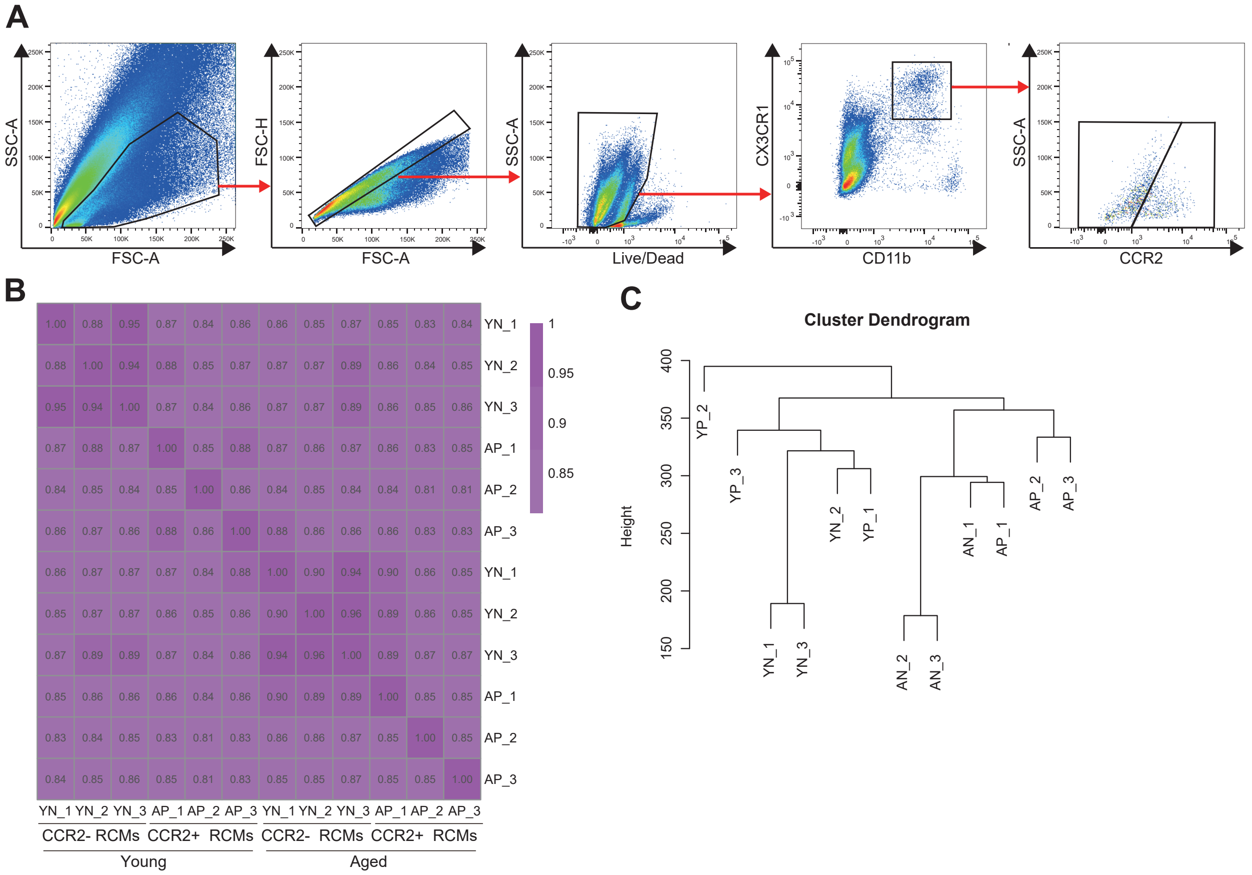

Suppl. Fig. 1 FACS sorting and SMART-sequencing quality control for cardiac macrophages from young and aged mice heart.

A, FACS gating strategy for CCR2⁻ and CCR2⁺ CRMs. **B**, Pearson correlation analysis between RNA-sequencing samples. **C**, Unsupervised cluster dendrogram between RNA-sequencing samples.

Supplementary Figure 2

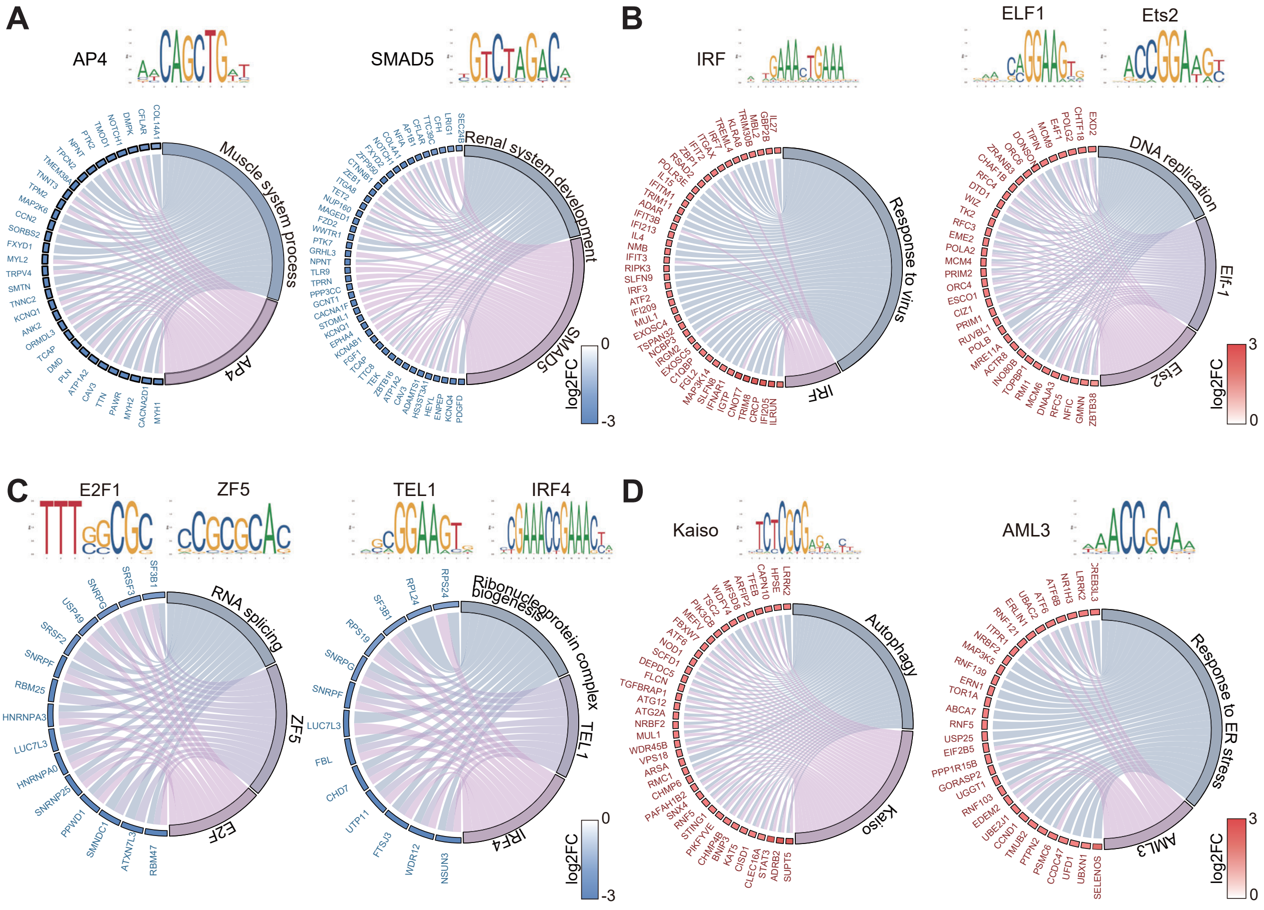

Suppl. Fig. 2 Identification of transcription factors for major biological processes in aging.

Transcription factors analysis for major enriched biologic processes in CCR2$^-$ CRMs in young mice **(A)**, CCR2$^-$ CRMs in aged mice **(B)**, CCR2$^+$ CRMs in young mice **(C)** and CCR2$^+$ CRMs in aged mice **(D)**.

Supplementary Figure 3

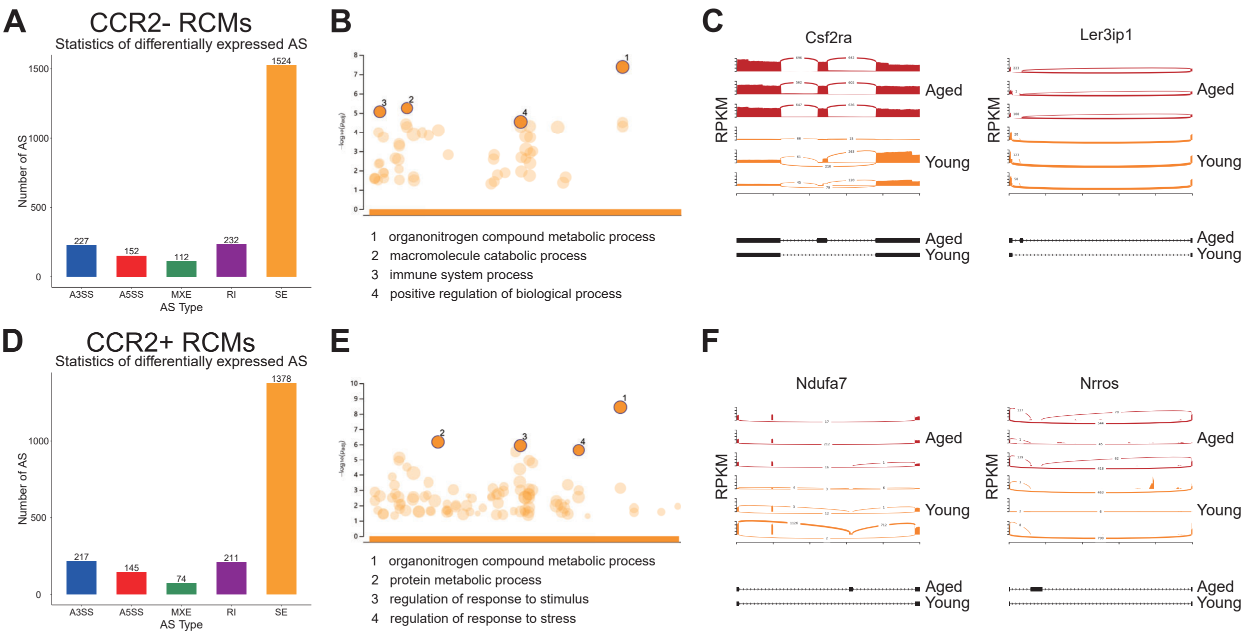

Suppl. Fig. 3 Alternative splicing analysis for CRMs in aging.

(A and D), Distribution of the five types of differentially expressed alternative splicing in aged CCR2$^-$ CRMs versus young CCR2$^-$ CRMs **(A)** and aged CCR2$^+$ CRMs versus young CCR2$^+$ CRMs **(D)**. A3SS, alternative 3' splice site. A5SS, alternative 5' splice site. MXE, mutually exclusive exons. RI, retained intron. SE, skipped exon. **(B and E)**, Gene ontologies enrichment for differentially expressed alternative splicing in CCR2$^-$ CRMs **(B)** and CCR2$^+$ CRMs **(E)** from aged mice versus young mice. **(C and F)** Schemes illustrate the representative alternative patterns in CCR2$^-$ CRMs **(C)** and CCR2$^+$ CRMs **(F)** from aged mice versus young mice.
